# Resolving Differences in Absolute Irradiance Measurements Between the SOHO/CELIAS/SEM and the SDO/EVE

**DOI:** 10.1007/s11207-014-0519-5

**Published:** 2014-04-01

**Authors:** S. R. Wieman, L. V. Didkovsky, D. L. Judge

**Affiliations:** University of Southern California Space Sciences Center, 835 Bloom Walk, Los Angeles, CA 90089 USA

**Keywords:** Absolute EUV irradiance, Calibration, SOHO/SEM

## Abstract

The *Solar EUV Monitor* (SEM) onboard SOHO has measured absolute extreme ultraviolet (EUV) and soft X-ray solar irradiance nearly continuously since January 1996. The *EUV Variability Experiment* (EVE) on SDO, in operation since April of 2010, measures solar irradiance in a wide spectral range that encompasses the band passes (26 – 34 nm and 0.1 – 50 nm) measured by SOHO/SEM. However, throughout the mission overlap, irradiance values from these two instruments have differed by more than the combined stated uncertainties of the measurements. In an effort to identify the sources of these differences and eliminate them, we investigate in this work the effect of reprocessing the SEM data using a more accurate SEM response function (obtained from synchrotron measurements with a SEM sounding-rocket clone instrument taken after SOHO was already in orbit) and time-dependent, measured solar spectral distributions – *i.e*., solar reference spectra that were unavailable prior to the launch of the SDO. We find that recalculating the SEM data with these improved parameters reduces mean differences with the EVE measurements from about 20 % to less than 5 % in the 26 – 34 nm band, and from about 35 % to about 15 % for irradiances in the 0.1 – 7 nm band extracted from the SEM 0.1 – 50 nm channel.

## Introduction

**Figure 1:**
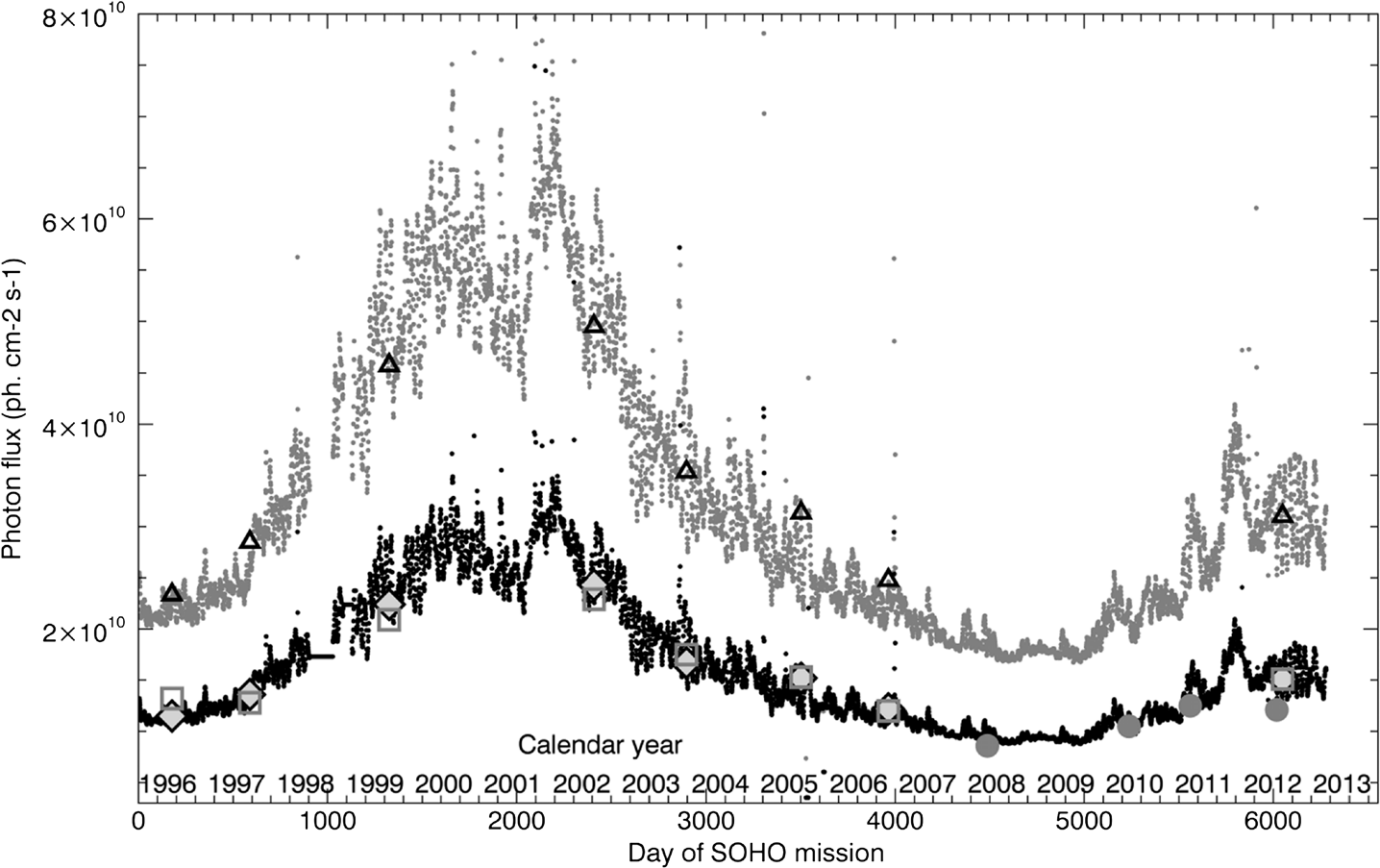
Photon flux time-series in the 26 – 34 nm band pass measured with the Version 3.1 SOHO/SEM first-order channels (small black circles) and in the 0.1 – 50 nm band pass measured with the SOHO/SEM zeroth-order channel (small gray circles). Sounding-rocket calibration measurements include RGIC full 5 – 57.5 nm band (triangles), SEM sounding-rocket clone 26 – 34 nm band (squares), RGIC scaled to 26 – 34 nm (diamonds), and EVE/ESP sounding-rocket clone (large gray circles).

Absolute solar irradiance measurements in the highly variable X-ray and EUV spectral ranges have both fundamental research value and practical utility and have become increasingly available during the past two decades, with EUV and soft X-ray irradiance instruments onboard multiple satellites, including the *Solar and Heliospheric Observatory* (SOHO; Harrison *et al.*, 1995; Hovestadt *et al.*, 1995), the *Solar Dynamics Observatory* (SDO; Woods *et al.*, 2012), the *Thermosphere Ionosphere Mesosphere Energetics Dynamics* (TIMED; Woods *et al.*, 2005), *Solar Radiation Climate Experiment* (SORCE; Woods and Rottman, 2005), ISS (Nikutowski *et al.*, 2011), PROBA2 (Dominique *et al.*, 2013), and GOES (Evans *et al.*, 2010). Among these, SOHO’s *Solar EUV Monitor*/*Charge, Element, and Isotope Analysis System* (SOHO/CELIAS/SEM; Judge *et al.*, 1998) 26 – 34 nm and 0.1 – 50 nm datasets (Figure 1) are unique in that they include high time-cadence (15 s), continuous (with the exception of the SOHO mission interruption of 1998) measurements that so far span more than 17 years and include two solar minima. Furthermore, the absolute calibration of the SEM data is maintained based on measurements from a long series of sounding-rocket flights (eight since the launch of SOHO, with the next scheduled for the summer of 2014) using a SEM clone instrument and a neon rare-gas ionization cell (RGIC) absolute detector. The SEM data are the source of the S10.7 solar EUV irradiance proxy (Tobiska, Bouwer, and Bowman, 2006; Bowman *et al.*, 2008), and they are widely used for inter-comparison with other EUV instrumentation (Thompson, McMullin, and Newmark, 2002; McMullin *et al.*, 2002; Woods *et al.*, 2005; Wieman, Judge, and Didkovsky, 2011; Didkovsky *et al.*, 2010b) and as a basis for validating EUV irradiance models (Chamberlin, Woods, and Eparvier 2007; Haberreiter 2012). Additionally, the SEM data are central to debates over whether and by how much solar EUV irradiance was lower during the minimum of solar cycles 23/24 than that of solar cycles 22/23. The SEM data suggest that the 26 – 34 nm irradiance was 15 % lower during the latter minimum (Didkovsky *et al.*, 2010a), an assertion that is supported by thermospheric data (Solomon *et al.* 2011), but not by ionospheric total electron content data (Lean *et al.* 2011). For these applications, and for continuation of the longstanding EUV record established by the SEM using newer EUV instrumentation, it is important to understand the instrumental and data processing factors that may affect the accuracy of the SEM absolute irradiances (SEM data are available at http://www.usc.edu/dept/space_science/).

The current period of overlap between the SOHO and SDO missions has provided a good opportunity to evaluate the accuracy of the SEM irradiance measurements based on comparisons with concurrent, independently calibrated measurements from SDO/EVE (Didkovsky *et al.* 2012; Hock *et al.* 2012; Woods *et al.* 2012). The EVE includes the *Extreme ultraviolet SpectroPhotometer* (ESP) channel, an instrument very similar to SOHO/SEM with a band pass nearly equivalent to the SEM 26 – 34 nm channel, as well as the *Multiple EUV Grating Spectrographs* (MEGS) channels, which provide high-resolution spectra in the 6 – 106 nm spectral range. Comparisons with EVE have shown that since the beginning of the SDO mission, SOHO/SEM Version 3.1 irradiances in the 26 – 34 nm band have been about 20 to 25 % higher (Didkovsky *et al.*, 2010b; Wieman, Judge, and Didkovsky, 2011) than both the ESP and the MEGS spectra integrated over 26 – 34 nm. The SOHO/SEM irradiances have been found to be higher by a similar amount in earlier comparisons with other EUV instruments, including the SOHO *Coronal Diagnostic Spectrometer* (CDS) in Thompson, McMullin, and Newmark (2002) and TIMED/SEE in Woods *et al.* (2005). For these earlier comparisons, the 20 % higher SOHO/SEM irradiance was within the combined calibration uncertainties (estimated uncertainties are 10 %, 20 %, and 25 % for SOHO/SEM, SOHO/CDS, and TIMED/SEE, respectively). However, the same cannot be said for the comparisons with SDO/EVE because of the relatively low estimated uncertainties of about 10 % for ESP (Didkovsky *et al.* 2012) and between 5 % and 20 % depending on wavelength for MEGS (Hock *et al.* 2012). Understanding these differences between the SOHO/SEM and SDO/EVE irradiances is one of the motivations for this study.

Since the latest release (Version 3.1) of the SOHO/SEM measurements we have obtained new calibration measurements of the SEM instrument response function and measurements of the time-dependent solar spectral distribution (*i.e*. reference spectrum), both of which are key parameters for calculating calibrated irradiances from the SEM raw data. The instrument response function of the SEM instrument onboard SOHO was measured pre-launch at the National Institute of Standards and Technology (NIST) Synchrotron Ultraviolet Radiation Facility (SURF III). More recently, however, a revised calibration procedure used for measuring the response function of the SEM sounding-rocket clone instrument at NIST revealed additional sensitivity of the SEM optical design to wavelengths outside of the nominal first-order 26 – 34 nm band pass. The nominally identical on-orbit SOHO/SEM most likely shares this out-of-band sensitivity, and we present in this work a SOHO/SEM response function that has been revised accordingly based on the SEM clone measurements (we refer to the SEM sounding-rocket clone instrument as the SEM clone and to the SEM instrument on orbit onboard SOHO as SOHO/SEM).

For much of the SOHO mission, measured solar spectral distributions in the EUV and soft X-ray range have not been available. As an alternative, the SOLERS22 spectrum (Woods *et al.* 1998), a single modeled solar spectrum, has been used to date in SOHO/SEM data processing (including Version 3.1). The SOLERS22 spectrum was adopted for processing SOHO/SEM data because it is widely known and available (McMullin *et al.* 2002) and because it represents a solar spectrum midway between solar minimum and solar maximum conditions and is therefore reliable over a broad range of activity levels (Judge *et al.* 2002). However, knowing the spectral distribution for deriving irradiance from broadband measurements is a long-standing problem (Wende 1972; Acton, Weston, and Bruner 1999), and it has been suggested that differences in the SOHO/SEM irradiances compared with those of other EUV instruments may be related to choice of reference spectrum (Thompson, McMullin, and Newmark, 2002; Woods *et al.*, 2005; Didkovsky *et al.*, 2010b; Wieman, Judge, and Didkovsky, 2011). In Wieman, Judge, and Didkovsky (2011), the SOHO/SEM data set was recalculated for the period of overlap with SDO using directly measured reference spectra from MEGS (*i.e*. measured concurrently with the corresponding SEM observations) in place of SOLERS22. This study showed that while the choice of reference spectrum does indeed have a significant effect on the calculated irradiance values, the differences between SEM and EVE cannot be explained based on reference spectrum alone.

The primary purpose of this study is to demonstrate that these differences can be largely resolved by reprocessing the SOHO/SEM data using both the more recently available SDO/EVE/MEGS reference spectra and the broader response function derived from the latest NIST calibration of the SEM clone. While only the SOHO/SEM data overlapping the period of SDO are addressed here, the new response function and the approach of using time-dependent reference spectra could be applied to the entire data set back to 1996. For reprocessing the earlier (*i.e*. prior to SDO) SOHO/SEM data a time-dependent reference spectrum will need to be established, and the SOHO/SEM time series presented here can serve as a standard against which to evaluate such alternative spectra. For example, reference spectra from proxy-based spectral models (*e.g*., FISM: Chamberlin, Woods, and Eparvier, 2007; or Solar2000/SIP: Tobiska, Bouwer, and Bowman, 2006) or from a combination of modeling and measurements (*e.g*., Del Zanna *et al.*, 2001; Thompson, McMullin, and Newmark, 2002) could be compared with the MEGS spectra based on how they affect the calculation of SEM irradiances for the period of overlap with SDO. The derivation of such pre-SDO reference spectra and recalculation of the complete SOHO/SEM time series back to 1996 is the subject of future work.

An overview of the SOHO/SEM instrument, including the data processing algorithms and details of the revised response function, is presented in Section 2 below. Although the SOHO/SEM algorithms have been reported previously in Judge *et al.* (1998) and McMullin *et al.* (2002) and have not been modified for this work beyond the substitution of the new response function and reference spectra, we present them here in an expanded form to show more explicitly their dependence on these two parameters. The SOLERS22 spectrum is compared with MEGS spectra for various levels of activity in Section 3. To validate the new response function and the use of MEGS measured reference spectra we present several comparisons between SEM and EVE in Section 4. Our conclusions based on these results and comments on how to apply this approach to recalculating SOHO/SEM irradiances for all of solar cycle 23 are reported in Section 5.

## SOHO/SEM Instrument

### Instrument Overview

The SOHO/CELIAS/SEM instrument design is described in detail in Hovestadt *et al.* (1995) and in Judge *et al.* (1998) and is described only briefly here. The SEM uses a highly stable freestanding transmission grating (Schattenburg and Anderson, 1990; Scime *et al.*, 1995) and radiation-hard silicon photodiode detectors (Funsten *et al.* 2004; Krumrey *et al.* 2000) with high efficiency in the EUV/soft X-ray spectral range. Aluminum thin-film filters, one freestanding at the entrance slit and one deposited on the surface of each of the three photodiode detectors, prevent the detection of visible light. Two of the detectors are positioned symmetrically in the grating diffraction pattern to detect photons in the 26 – 34 nm band pass diffracted in the +1 and −1 orders, and the third detector is positioned to detect the zeroth order with its band pass constrained by the aluminum thin films to effectively 0.1 – 50 nm.

The absolute response function of the SEM instrument (with the exception of the soft X-ray portion of the 0.1 – 50 nm channel) was measured prior to the launch of SOHO at NIST SURFIII (Furst, Graves, and Madden, 1993; Vest *et al.*, 1999) on beam-line 9, which is equipped with a monochromator that allows the channel response functions to be measured with 1 nm resolution over a spectral range from about 15 to 49 nm. The response function of the zeroth-order channel for soft X-ray wavelengths shorter than 15 nm is modeled based on photoabsorption and transmission values for the relevant materials found in Henke, Gullikson, and Davis (1993).

Calibration of the SEM instrument has been maintained over the course of the SOHO mission through periodic sounding-rocket measurements made using a clone of the SEM instrument (Judge *et al.*, 2002) and a RGIC absolute detector (Carlson *et al.*, 1984; Ogawa and Judge, 1986). Figure 1 shows the time series of SEM photon flux measurements (SEM Version 3.1) in the 26 – 34 nm and 0.1 – 50 nm band passes for the SOHO mission thus far, plotted with EUV measurements from the SEM calibration underflights, as well as from a similar wavelength channel on the EVE/ESP clone sounding-rocket instrument. RGIC measurements have been scaled to the 26 – 34 nm band pass based on the SOLERS22 solar reference spectrum for comparison with the SEM first-order channels, but remain in their native ≈ 5 – 57 nm band pass (constrained by the photoabsorption range for neon) for comparison with the zeroth-order channel.

Some loss of sensitivity of the SOHO/SEM is evident from the sounding-rocket measurements and is attributed to the buildup and subsequent UV-photon-driven polymerization of hydrocarbons on the aluminum thin-film filters. A finite contaminant source, present with an exponentially diminishing pressure, is assumed. The degradation is accordingly modeled as a contaminant layer that grows in thickness, *τ*, with time according to

1 $$ \tau (t) = a + b \cdot \mathrm{e}^{-t/c}, $$

where *t* is time from the beginning of the mission and *a*, *b*, and *c* are parameters that are determined to minimize discrepancies between sounding-rocket and corresponding on-orbit measurements. The exact composition of the contaminant is unknown, but pure carbon is used to define wavelength dependence in the degradation model and has resulted in good agreement with the sounding-rocket measurements, as shown in Figure 1. The degradation factor is applied as a wavelength-dependent transmission value between 0 and 1 determined from carbon photoabsorption cross-sections, *σ*_C_(*λ*), as

2 $$ f_{\mathrm{degrad}}(\lambda,t) = \mathrm{e}^{- \sigma_{\mathrm{C}}\cdot\tau}. $$

### SEM 26 – 34 nm Channel

#### Channel Response Function

**Figure 2:**
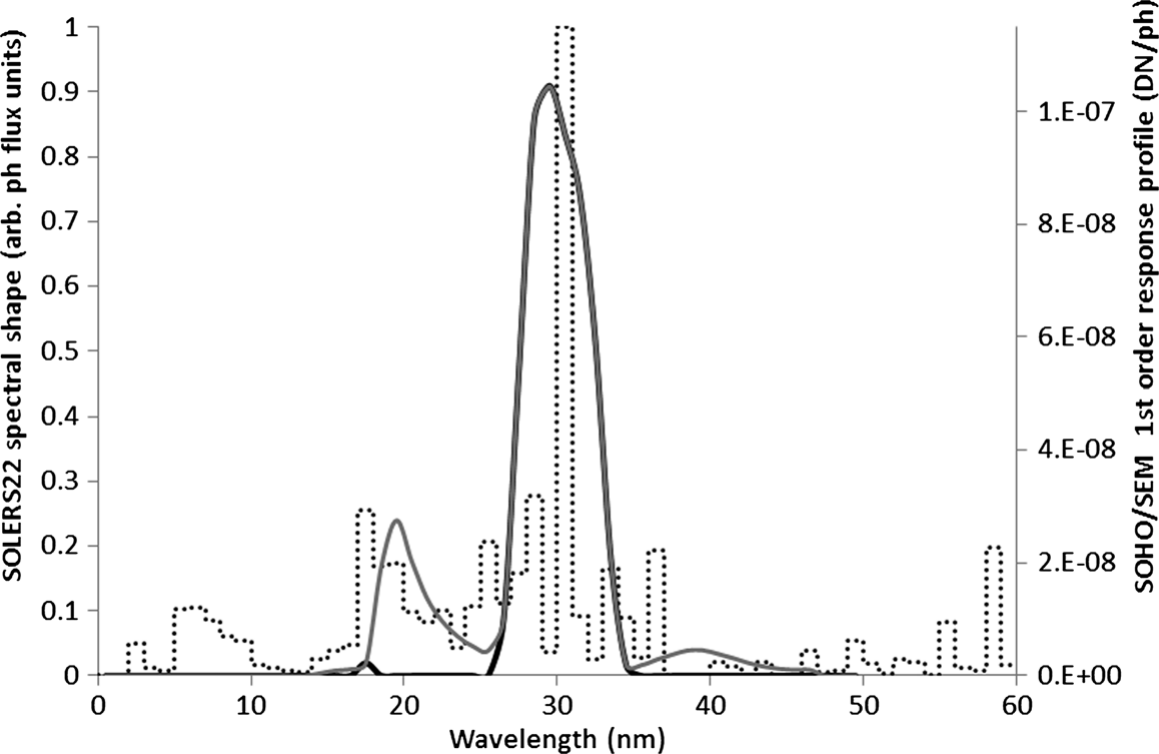
SOHO/SEM first-order response function used for irradiance calculation in Version 3.1 (black curve partially obscured by the gray curve, which is identical over the wavelength range from 26 to 34 nm) and in the updated version reported here (gray line). The SOLERS22 spectrum is shown for reference (dotted line).

The Version 3.1 response function of the SOHO/SEM first-order (26 – 34 nm) channel measured prior to launch is plotted as a black line in Figure 2. The SOLERS22 reference solar spectrum (dashed line) is also included for comparison. For the early SOHO/SEM and SEM clone calibrations a portion of the housing was removed as a precaution to prevent excessive mechanical stresses in the free-standing grating due to gas pressure gradients that would otherwise develop across the grating during the repeated evacuation and venting of the beamline test chamber with each calibration. During a 2007 post flight calibration of the SEM sounding-rocket clone instrument (by which time the mechanical stability of the gratings had been established with greater certainty) the complete housing was used and was found to produce two additional peaks in the first-order response function, one on either side of the primary 26 – 34 nm band pass. This effect is attributed to the grazing incidence reflection of longer wavelength photons (and shorter wavelength photons diffracted in the second order) off the inner surface of the housing and into the first-order detectors. For the revised version of the SEM irradiances reported here, modified first-order response functions (the +1 and −1 order response functions are slightly different) have been modeled for the SOHO/SEM based on the SEM clone calibration measurements taken with the complete housing. The modeled SOHO/SEM first-order response functions, shown for one of the channels as a gray line in Figure 2, is obtained by substituting the SEM clone response values (the average of the two, +1 and −1, first-order channels of the SEM clone) from 13 – 26 nm and from 34 – 47 nm into the SOHO/SEM response function after scaling the values by the ratio of the peak efficiency for SOHO/SEM over that for the SEM clone.

#### Algorithm for Converting First-Order Raw Count Rates to Photon Flux Values

We show here first the algorithm for determining 26 – 34 nm irradiance from SEM count rate data based on the SOLERS22 reference spectrum. Our approach for substituting the MEGS reference spectrum is described in Section 3.

The SEM 26 – 34 nm flux, Φ_SEM1_ (expressed in photon flux units [ph cm^−2^ s^−1^] in Version 3.1), is the mean of the two symmetric plus and minus first diffraction-order channels. The flux in each channel is calculated from its detector’s DN according to

3 $$ \Phi_{\mathrm{SEM}1} = k \frac{\mathrm{DN}_{\mathrm{SEMch}1} - I_{\mathrm{bkgrd}}}{ \frac{A \cdot f_{1~\mathrm{AU}} \cdot \int_{\lambda_1}^{\lambda_{2}} \eta_{1} \cdot \phi_{\mathrm{S}22} \cdot f_{\mathrm{degrad}}\,\mathrm{d}\lambda}{\int_{\lambda_1}^{\lambda_{2}} \phi_{\mathrm{S}22}\,\mathrm{d}\lambda}}, $$

where *k*, defined below, is a correction for the SEM sensitivity band that extends slightly beyond 26 – 34 nm (including second-order contributions from wavelengths near 17 nm), *I*_bkgrd_ is a background signal due to diode/electrometer dark current and residual light leaks, DN_SEMch1_ is a first-order channel raw count rate, *A* is the entrance aperture area, *η*_1_ is the SEM first-order channel efficiency from NIST calibration (described above in Section 2.4.1), *ϕ*_S22_ is the reference spectrum solar flux (*i.e*. SOLERS22 for SOHO/SEM Version 3.1), *f*_degrad_ is a time- and wavelength-dependent degradation factor (described above in Section 2.1), *f*_1 AU_ is a correction to normalize observations to a distance of 1 AU, and *λ*_1_ and *λ*_2_ delineate the range of wavelengths over which the SEM first-order channel is sensitive, which extends slightly beyond the reported 26 – 34 nm band. The specific values of *λ*_1_ and *λ*_2_ vary slightly depending on which of the first-order channels (*i.e*. +1 or −1 diffraction order) is considered and whether the pre-flight or modified response function is used.

The SEM response function, *η*_1_, as well as the degradation term, *f*_degrad_, are both functions of wavelength and thus it is necessary to determine a weighted mean value for these terms with the weight factors equal to the relative intensity of spectral bins within a reference solar spectrum. The SOLERS22 reference spectrum has been used throughout the SOHO mission including the most recent release, Version 3.1. Additionally, the value *k*, which corrects for SEM first-order sensitivity that extends slightly beyond the reported 26 – 34 nm band pass and includes some second-order contribution from wavelengths near 17 nm, is also determined based on the SOLERS22 reference spectrum according to

4 $$ k = \frac{\int_{26~\mathrm{nm}}^{34~\mathrm{nm}} \phi_{\mathrm{S}22}\, \mathrm{d}\lambda}{\int_{\lambda_1}^{\lambda_{2}} \phi_{\mathrm{S}22}\,\mathrm{d}\lambda}. $$

Thus, the weighted average efficiency, the weighted average degradation factor, and the second-order/out-of-band correction are all dependent on the spectral shape, but not the absolute irradiance values of the reference spectrum.

Equation (3) can be simplified by substituting the right-hand side of Equation (4) in for *k*, leaving

5 $$ \Phi_{\mathrm{SEM}1} = \frac{ ( \mathrm{DN}_{\mathrm{SEMch}1} - I_{\mathrm{bkgrd}} ) \int_{26~\mathrm{nm}}^{34~\mathrm{nm}} \phi_{\mathrm{S}22} \,\mathrm{d}\lambda}{A \cdot f_{1~\mathrm{AU}} \cdot \int_{\lambda_1}^{\lambda_{2}} \eta_{1} \cdot \phi_{\mathrm{S}22} \cdot f_{\mathrm{degrad}} \,\mathrm{d}\lambda}. $$

### SEM 0.1 – 50 nm Channel

#### Channel Response Function

**Figure 3:**
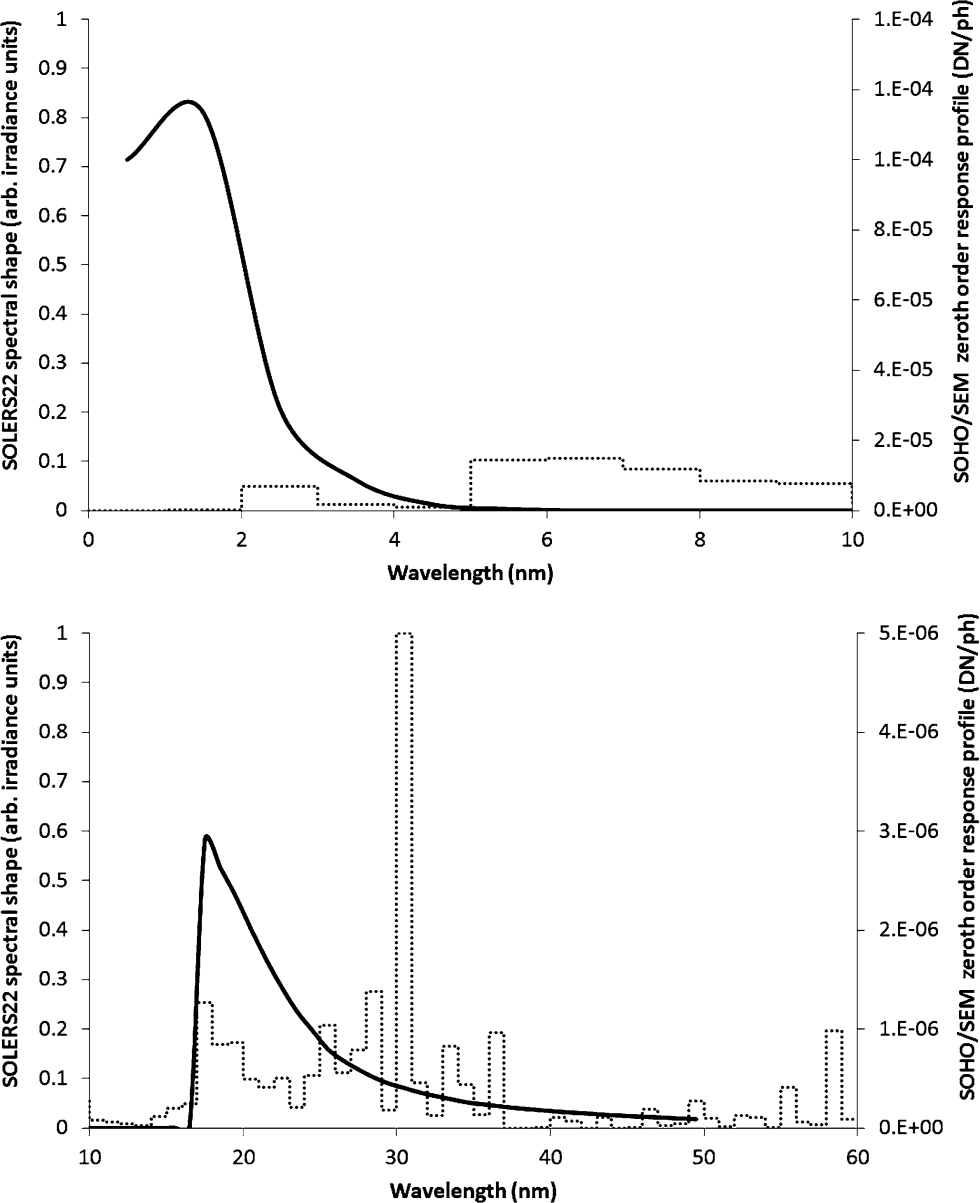
Soft X-ray (top panel) and EUV (bottom panel) portions of the SOHO/SEM zeroth-order response function used for irradiance calculations in both Version 3.1 and in the updated version reported here (black line). The SOLERS22 spectrum is shown for reference (dotted line, units are arbitrary but consistent between the top and bottom panels).

The SOHO/SEM 0.1 – 50 nm response function is constrained spectrally by the aluminum thin-film filters in front of the entrance slit and deposited on the zeroth-order detector. The profile, shown in Figure 3, is non-uniform over the reported spectral range with most of the signal coming from the EUV band between 15 and 50 nm and the soft X-ray band shorter than 5 nm. Unlike the first-order channel detectors, the zeroth-order detector is not within the path of photons reflected at grazing incidence off of the inside of the SEM housing and therefore the zeroth-order response function remains nominally the same whether or not the complete housing is installed. Thus, for the work reported here we continue to use the original zeroth-order response function that has been used throughout the SOHO/SEM mission.

#### Algorithm for Converting Zeroth-Order Raw Count Rates to Photon Flux Values

We show here the algorithm for determining 0.1 – 50 nm irradiance from SEM count rate data based on the SOLERS22 reference spectrum. Our approach for substituting the MEGS reference spectrum is described in Section 3.

Solar soft X-ray fluxes are known to have greater variability with time than EUV fluxes, but because the SOLERS22 spectrum is fixed with time it does not capture this relative variability. Thus it is not expected (Judge *et al.* 2002) that SOLERS22 provides a reliable reference spectrum over the full SEM zeroth-order (0.1 – 50 nm) range of sensitivity for all levels of solar activity. To account for changes in the relative contribution of soft X-ray versus EUV photons over time, different time-dependent scaling factors, *α* and *κ*, are applied to the soft X-ray (0.1 – 5 nm) and EUV (5 – 50 nm) portions of the adopted reference spectrum, respectively,

6 $$ \phi_{\mathrm{SEM}0} ( \lambda ) = \begin{cases} \alpha\cdot \phi_{\mathrm{S}22} ( \lambda ),& \lambda=0.1\,\mbox{--}\,5~\mathrm{nm},\\ \kappa\cdot \phi_{\mathrm{S}22} ( \lambda ),& \lambda=5\,\mbox{--}\,50~\mathrm{nm}, \end{cases} $$

where *ϕ*_S22_ is the SOLERS22 reference spectrum for Version 3.1, and the reported SEM zeroth-order 0.1 – 50 nm photon flux, Φ_SEM0_, is the integral of the scaled reference spectrum,

7 $$ \Phi_{\mathrm{SEM}0} = \int_{0.1}^{50} \phi_{\mathrm{SEM}0} \,\mathrm{d}\lambda. $$

The scaling factor for the EUV wavelengths, *κ*, is calculated such that the constructed spectrum, when integrated over the 26 – 34 nm band pass, is equal to Φ_SEM1_, the flux measured in the first-order channels according to

8 $$ \kappa= \frac{\Phi_{\mathrm{SEM}1}}{\int_{26~\mathrm{nm}}^{34~\mathrm{nm}} \phi_{\mathrm{S}22} \,\mathrm{d}\lambda}. $$

The EUV portion of the reference spectrum scaled by *κ*, and the zeroth-order response function, *η*_0_(*λ*) for *λ*=5 – 50 nm, provide the estimated portion of the SEM zeroth-order raw count rate, DN_SEMch0_, which is related to EUV photons. The remaining portion of the zeroth-order effective counts (*i.e*. related to soft X-ray photons) are then used with the response function, *η*_0_(*λ*) for *λ*=0.1 – 5 nm, to determine the scaling factor, *α*, according to

9 $$ \alpha= \frac{ ( \frac{\mathrm{DN}_{\mathrm{SEMch}0} - I_{\mathrm{bkgrd}0}}{f_{1~\mathrm{AU}} \cdot A} ) - \int_{5~\mathrm{nm}}^{50~\mathrm{nm}} \kappa \cdot \phi_{\mathrm{S}22} \cdot \eta_{0} \cdot f_{\mathrm{degrad}} \,\mathrm{d}\lambda}{\int_{0.1~\mathrm{nm}}^{5~\mathrm{nm}} \phi_{\mathrm{S}22} \cdot \eta_{0} \cdot f_{\mathrm{degrad}} \,\mathrm{d}\lambda}, $$

where *f*_degrad_ is based on the same carbon-growth model described in Section 2.1 and used for calculating degradation in the first-order channels.

## Solar Reference Spectra

While SOLERS22 does not capture variations in the solar spectral distribution with time, continuous time-varying spectra are available from the SDO/EVE instrument suite (Hock *et al.* 2012; Woods *et al.* 2012). The EVE provides solar spectral irradiance measurements in the extreme ultraviolet and soft X-ray ranges that are unprecedented in terms of spectral resolution, time cadence, accuracy, and precision. Irradiance spectra over the 6 – 106 nm range are obtained with 0.2 Å resolution using the EVE/MEGS channels. Calibration of the EVE solar spectral irradiance measurements is maintained through periodic sounding-rocket measurements with an EVE clone instrument. Additionally, EVE has provisions for in-flight measurements of degradation related to filter contamination, change in detector sensitivity, and dark current (Didkovsky *et al.* 2012; Hock *et al.* 2012). We thus believe the EVE/MEGS measured spectra are currently the most reliable source of reference spectra to use for processing SOHO/SEM data.

The high-resolution MEGS spectra cover the entire range of SOHO/SEM first-order channel sensitivity, so these spectra, *ϕ*_MEGS_(*t*,*λ*), can be substituted directly into the first-order irradiance [Equation (3)] in place of *ϕ*_S22_(*λ*). Because the MEGS spectra do not cover the soft X-ray range shorter than 6 nm, for the zeroth-order SOHO/SEM channel a different reference spectrum must be used to cover this short wavelength spectral range. Since measured soft X-ray spectra are not readily available, for this work we continue to use the SOLERS22 for this purpose and adopt the following scaled reference spectrum [in place of Equation (6) above] for calculating zeroth-order irradiances

10 $$ \phi_{\mathrm{SEM} 0} ( \lambda ) = \begin{cases} \alpha\cdot \phi_{\mathrm{S}22} ( \lambda ), & \lambda=0.1\,\mbox{--}\,5~\mathrm{nm},\\ \kappa\cdot \phi_{\mathrm{S}22} ( \lambda ), & \lambda=5\,\mbox{--}\,7~\mathrm{nm},\\ \kappa\cdot \phi_{\mathrm{MEGS}} (t, \lambda ), & \lambda=7\,\mbox{--}\,50~\mathrm{nm}. \end{cases} $$

Although the shortest wavelength included in the SDO/EVE spectra is at 6 nm, we use a lower boundary of 7.0 nm as a convention to accommodate alternate irradiance conversion algorithms that use measurements in the nominal 0.1 – 7 nm carbon-titanium filter band-pass (*e.g*. SDO/EVE/ESP quad diode, TIMED/SEE/XPS) to derive soft X-ray reference spectra. Such algorithms are beyond the scope of this work, and because solar irradiance is low in this range the slight shift of this boundary has little effect on the irradiance calculation.

**Figure 4:**
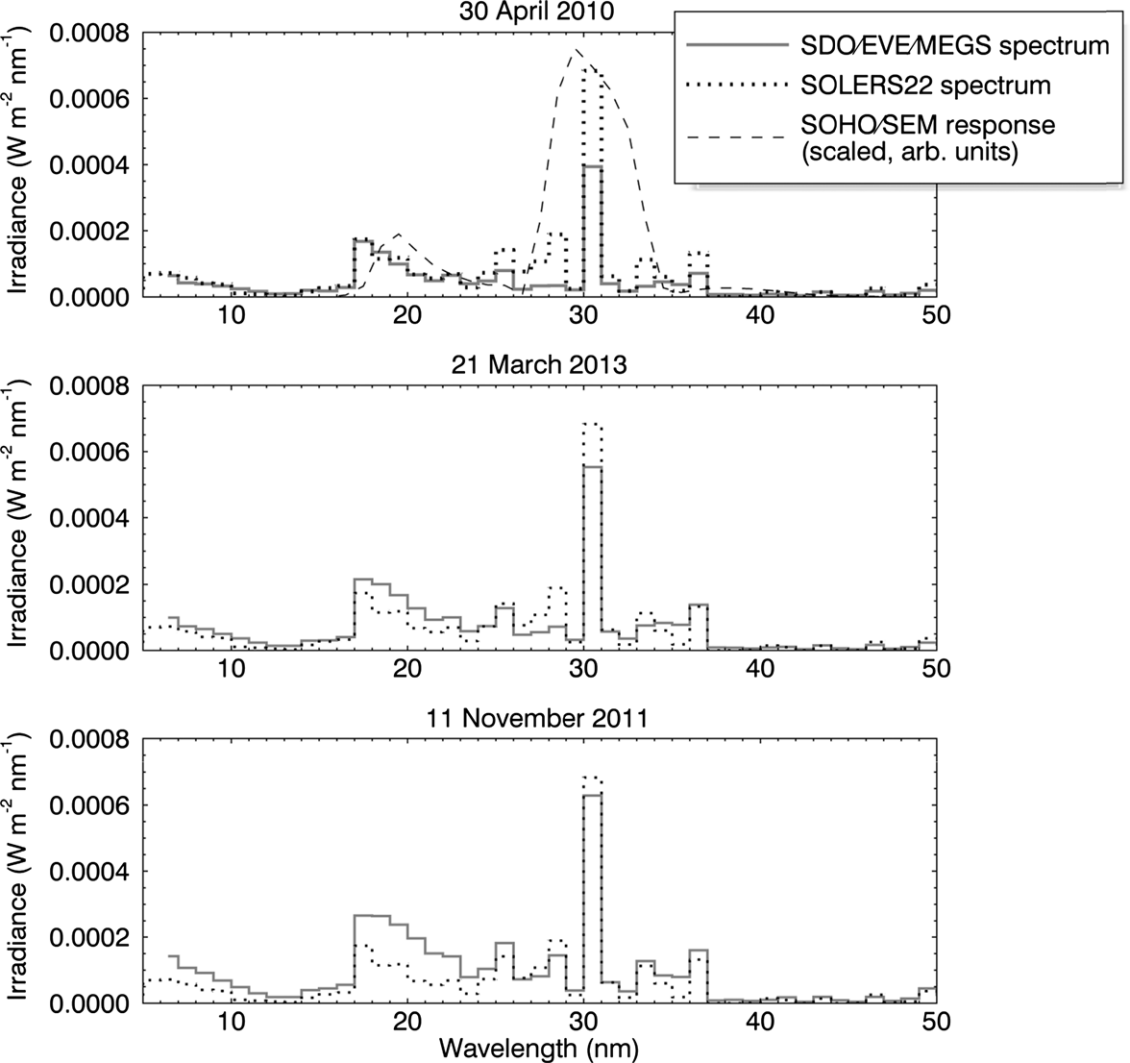
A comparison of the SOLERS22 fixed reference spectrum (dotted line) to measured SDO/EVE reference spectra (black line) for low (30 April 2010, top panel), medium (21 March 2013, middle panel), and high (11 November 2011, bottom panel) levels of solar activity – the SDO/EVE spectra shown are the daily average spectra for the specified day. In the top panel the SOHO/SEM first-order response function (dashed line) is shown for reference. Time-independent, characteristic differences exist between the spectra including a smaller contribution from the wavelength bins centered on 28.5 nm and 30.5 for the EVE/MEGS spectrum for all activity levels.

In Figure 4, the SOLERS22 reference spectrum is compared with daily average SDO/EVE Version 2 spectra for days of low (top panel), moderate (middle panel), and high (bottom panel) levels of activity. Moreover, we show in the top panel the SOHO/SEM updated response function for one of the SOHO/SEM first-order channels (scaled by an arbitrary factor to fit the plot). In addition to time dependence, there are some characteristic differences between the EVE spectra and the SOLERS22 spectrum. For example, for all activity levels, the wavelength bins centered on 28.5 nm and 30.5 (which include the Fe xv coronal and He ii transition region emission lines, respectively) make a lesser contribution to the EVE spectral distribution than they do for the SOLERS22 spectrum. When substituting the EVE spectra for SOLERS22 in the irradiance calculation, these differences affect the weighted average instrument response and degradation factor, and the *k* correction factor in Equation (3).

## Comparisons Between SOHO/SEM and SDO/EVE

We have performed several comparisons between SOHO/SEM and SDO/EVE measurements to validate the revised response function and the use of MEGS-measured reference spectra. SOHO/SEM irradiance values determined using this approach (referred to as updated SOHO/SEM irradiances) are also compared with Version 3.1 irradiances based on how well each data set agrees with the EVE measurements.

### Comparisons of the 26 – 34 nm Daily Average Time Series

**Figure 5:**
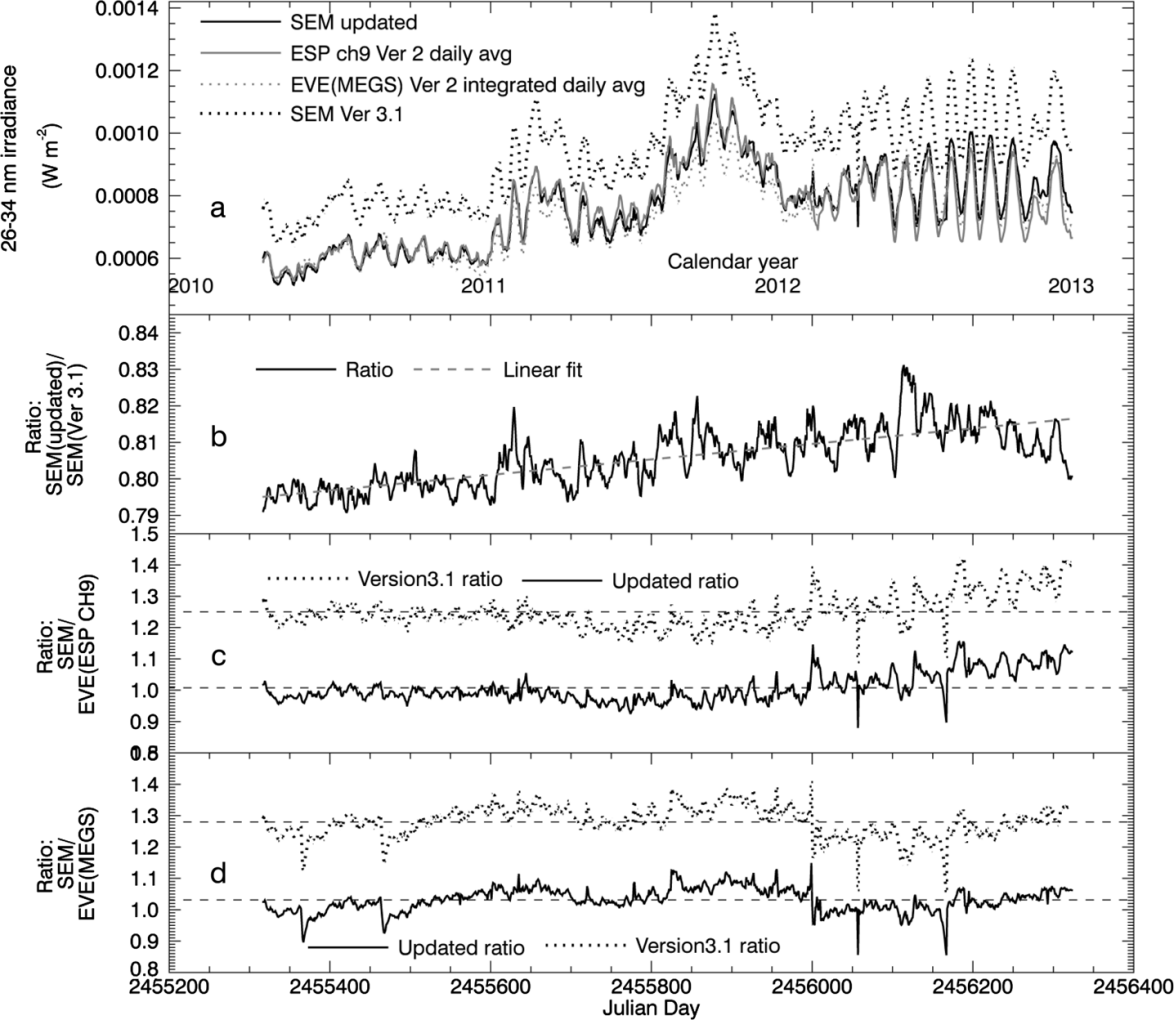
(a) A comparison of the updated SOHO/SEM 26 – 34 nm irradiance time-series (black curve) with SOHO/SEM Version 3.1 (dotted black curve), SDO/EVE/MEGS (dotted black curve) Version 2 spectra integrated over the 26 – 34 nm band pass, and EVE ESP ch9 (gray curve). (b) Ratio of the updated SOHO/SEM daily average irradiances over those of SOHO/SEM Version 3.1. (c) Ratios of the SOHO/SEM Version 3.1 daily average irradiances over the daily average SDO/EVE/ESP ch9 irradiances compared with similar ratios based on the updated SOHO/SEM. (d) Ratios of the SOHO/SEM Version 3.1 daily average irradiances over the daily average integrated SDO/EVE/MEGS spectra compared with similar ratios based on the updated SOHO/SEM. Mean ratio levels are shown as dashed lines in panels (c) and (d). The broader response function and the use of MEGS reference spectra for the updated SOHO/SEM irradiances result in significantly better agreement with both SDO/EVE channels. Divergence between the SOHO/SEM and SDO/EVE/ESP ch9 irradiances in 2012 could be related to higher sensitivity of the SEM to energetic particles than ESP or to errors related to using daily average reference spectra on days with high solar activity (see text for details).

Figure 5 compares the updated SOHO/SEM and Version 3.1 first-order (26 – 34 nm) daily average irradiances with both the MEGS and ESP channels of EVE. The SEM Version 3.1 data, which are normally published in photon flux units (photons cm^−2^ s^−1^), have been converted into irradiance units (W m^−2^) using the SOLERS22 reference spectrum to describe the energy distribution within the SEM band. For these comparisons the EVE/MEGS Version 2 *absolute* spectra are integrated over the 26 – 34 nm band in contrast to their use as a reference spectrum in SOHO/SEM data processing, for which they are normalized and only provide information about the relative spectral distribution. The comparison with EVE/ESP uses ESP Version 2 data from channel 9, which measures the 26.7 – 33.8 nm band pass. The ESP irradiances are scaled to account for the slight difference in band pass by a factor equal to the ratio of the SDO/EVE/MEGS integrated irradiances for the two band passes (the SEM band over the ESP band) for the corresponding day, according to

11 $$ \Phi_{\mathrm{ESP}} ' = \Phi_{\mathrm{ESP}} \cdot \frac{\int_{26}^{34} \phi_{\mathrm{MEGS}} \,\mathrm{d}\lambda}{ \int_{26.7}^{33.8} \phi_{\mathrm{MEGS}} \,\mathrm{d}\lambda}. $$

The daily ratio values in Figure 5b for the updated SOHO/SEM over Version 3.1 range from about 0.79 to about 0.83 over the time series shown and depend on the MEGS spectral distribution for the corresponding day. Solar cycle variability of these distributions results in a slight long-term trend in the ratio values apparent from the slope of the linear fit of about 10^−5^ day^−1^. Since the only time-dependent difference between the updated and Version 3.1 algorithms is the change in reference spectrum with solar-activity level, this trend is expected to reverse with the transition to the solar cycle 24 – 25 minimum. For the SOLERS22 reference spectrum, irradiance for the 30.4 nm He ii line is characteristically higher than for the MEGS spectra. Since this wavelength is near the peak of the SEM response function, the use of SOLERS22 results in a higher value for the weighted mean instrument response and thus lower values for the calculated irradiance according to Equation (3). However, due to the broader SEM response function, the updated SEM irradiances include a larger correction [*i.e*. lower *k* values based on Equation (4)] for the SEM signal outside of the reported SEM 26 – 34 nm band pass. The latter effect is more significant and results in the approximately 20 % decrease for the updated irradiance values, which makes them agree much better with both the integrated MEGS spectra and the ESP ch9 irradiances (compare ratios in Figures 5c and 5d). Mean ratios are reduced from about 1.25 to about 1.01 for the comparison with ESP ch9 and from about 1.27 to about 1.03 for the comparison with MEGS.

An apparent long-term trend in the SEM over ESP ratios in Figure 5c suggests that both SOHO/SEM versions are showing a greater increase with solar cycle 24 than EVE/ESP ch9. However, this trend is not consistent throughout the entire SDO mission. For example, linear fits to the ratios over the first half of the mission suggest an opposite trend. The source of such trends is not yet clear. One possible explanation is that the inaccuracies introduced by using daily average reference spectra to calculate irradiance for days in which significant changes in spectral distribution occur are not likely to be the same for SOHO/SEM as they are for EVE/ESP since the two instruments have different response functions. A second possibility is that the divergence is related to the higher susceptibility of the SOHO/SEM instrument to signal contamination due to energetic particles since the SEM instrument housing is much thinner and more easily penetrated by lower energy particles (Didkovsky *et al.* 2007) than that of EVE/ESP. The divergence between the SOHO/SEM and EVE/ESP data sets appears to increase with the transition from lower to higher levels of solar activity, which is consistent with both of these explanations. A third source of divergence could be related to changes in dark count rates for one or both of the instruments. For SOHO/SEM the dark count rates are assumed to be stable and for most of the EVE/ESP Version 2 data a dark-count model that only accounts for changes in temperature is used, yet in-flight dark count measurements with EVE/ESP have shown evidence of small changes in dark-count rates with both temperature and time.

Sharp drops followed by gradual rises in the SEM/EVE (MEGS) ratios of Figure 5d (*e.g*., on Julian days 2 455 364, 2 455 463, and 2 455 999) are due to abrupt increases of EVE/MEGS irradiance values following CCD bake-outs performed to restore sensitivity in the MEGS B channel.

### Comparison of 26 – 34 nm High Time-Cadence Measurements Under Flare Conditions

**Figure 6:**
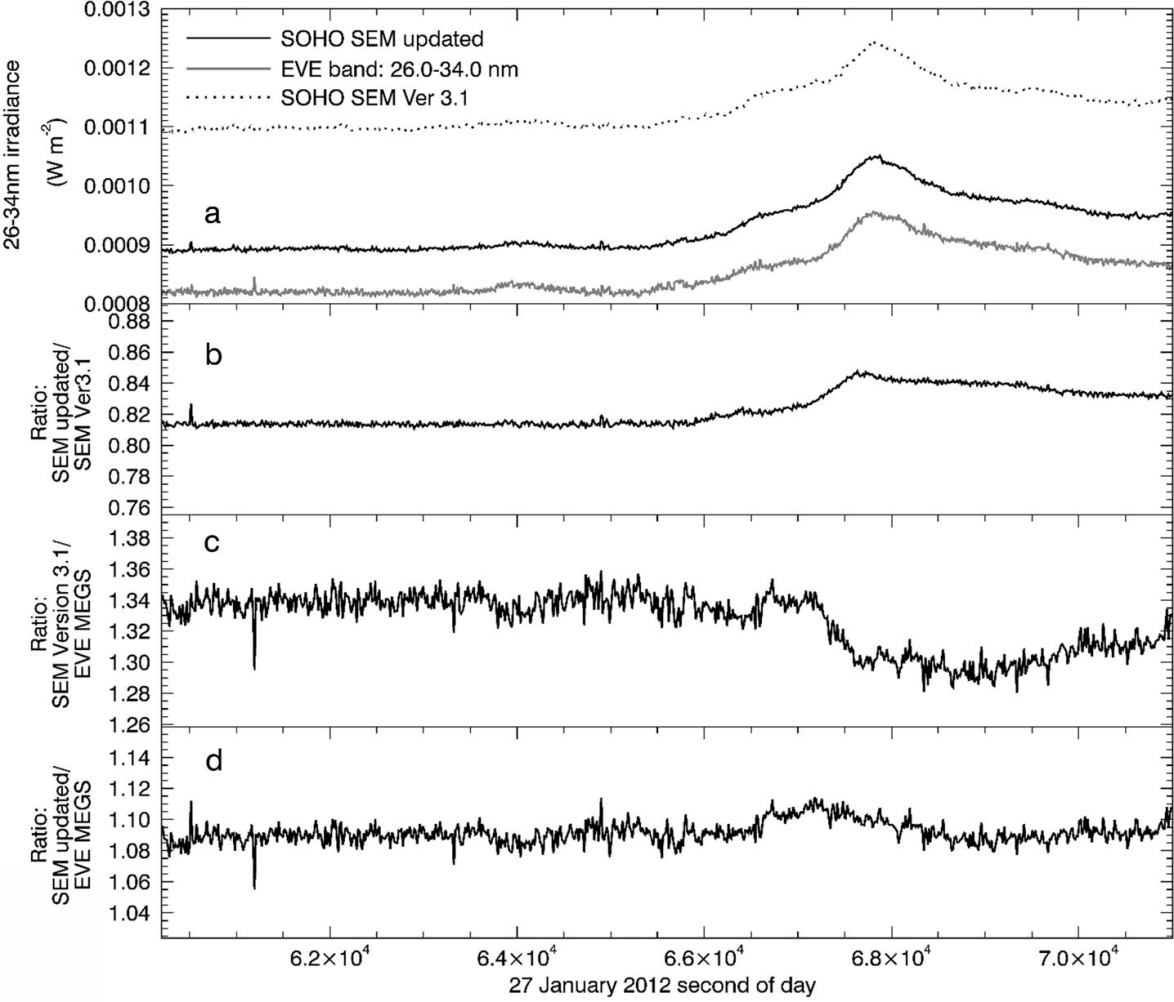
(a) A comparison of the updated SOHO/SEM 26 – 34 nm irradiance time-series (black curve) with SOHO/SEM Version 3.1 (dotted curve), and SDO/EVE/MEGS Version 2 spectra integrated over the 26 – 34 nm band pass with high time-resolution during the X1.7, N33W85 solar flare of 27 January 2012. (b) The ratios of the updated SOHO/SEM irradiances over those of SOHO/SEM Version 3.1 vary by several percent over the course of the flare due to the rapid change of solar spectral distribution during the flare. (c) Ratios of the SOHO/SEM Version 3.1 irradiances over the integrated SDO/EVE/MEGS Version 2 spectra deviate significantly from their pre-flare values, while ratios of the daily average updated SOHO/SEM over the daily average integrated SDO/EVE/MEGS spectra (d) show little change.

Because the updated SOHO/SEM irradiance measurements are based on time-dependent reference spectra, they should be less susceptible to loss of accuracy caused by rapid changes in solar spectral distribution that occur during a solar flare than the Version 3.1 measurements. They are therefore expected to be better correlated with MEGS integrated spectra under such conditions. For example, in Figure 6 updated and Version 3.1 SOHO/SEM measurements are compared with MEGS spectra integrated over 26 – 34 nm for a period of 3 h surrounding a 27 January 2012 X-class solar flare (X1.7, N33W85). The time cadence for the measurements is 10 s for the MEGS spectra and 15 s for the SOHO/SEM irradiances, thus near real-time (*i.e*. with an offset of 5 s or less) reference spectra are available for the SOHO/SEM irradiances calculated using our updated approach. Near the peak of the flare, the ratio of updated over Version 3.1 SOHO/SEM irradiance in Figure 6b shifts rapidly by about 4 % compared with its pre-flare level. During this time, the updated SOHO/SEM values that are calculated using the time-varying reference spectra track the EVE/MEGS integrated spectra much more closely than Version 3.1. The ratio of Version 3.1 SOHO/SEM irradiances over MEGS integrated irradiance shifts by nearly 5 % (Figure 6c) at the flare peak, while the ratio for the updated irradiances shifts by only about 1 % or 2 % (Figure 6d). This small shift is possibly caused by the remaining inaccuracy in the SOHO/SEM response function for which the out-of-band sensitivity was not measured directly, but derived from measurements of the SEM clone. Nonetheless, the relative consistency of the ratio throughout the course of the flare suggests that the updated parameters are a significant improvement over those from Version 3.1.

**Figure 7:**
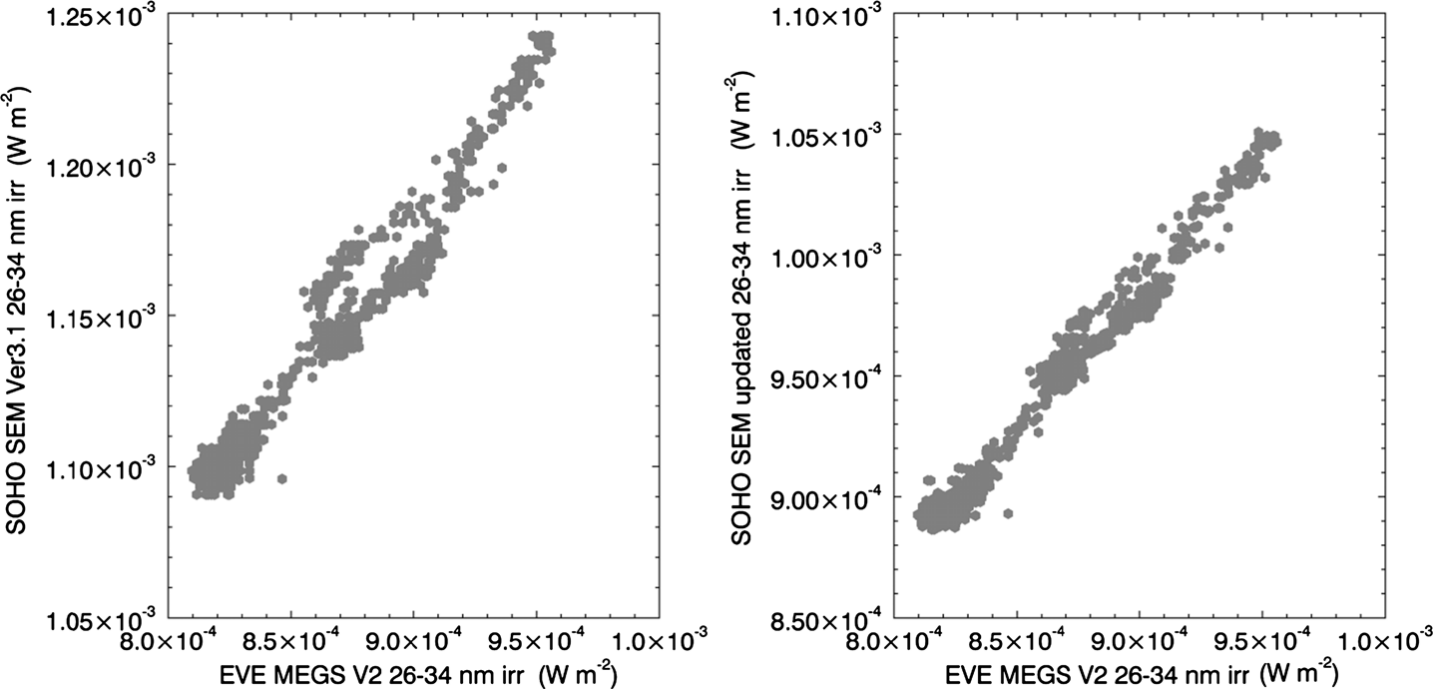
Correlations between EVE/MEGS spectra integrated over the 26 – 34 nm band and measurements from the SOHO/SEM 26 – 34 nm channels. SOHO/SEM Version 3.1 irradiances are shown in the left panel and those calculated using MEGS high time-cadence spectra and the revised response function are shown in the right panel. The correlation includes high time-cadence data over the 3-h period shown in Figure 6. Correlation coefficients are 0.98 for the SOHO/SEM Version 3.1 data and 0.99 for the updated SOHO/SEM data.

In Figure 7 the correlation between the two versions of SOHO/SEM irradiances and the MEGS integrated spectra are shown for the same 3-h time interval shown in Figure 6. The correlation between Version 3.1 and MEGS in the left panel of Figure 7 is already quite good (correlation coefficient, *r*_cor_=0.98) and leaves room for only modest improvement, but the updated version (right panel, *r*_cor_=0.99) does result in such an improvement. While this improvement is small, it is consistent throughout the period of mission overlap. For ten out of the 14 X-class flares occurring during this period the updated SOHO/SEM data result in moderately higher correlation coefficients than Version 3.1, and for the remaining four the correlation coefficients are equal. Abnormally low correlation coefficients were found for some flares (*e.g*., for the 23 October 2012 flare, *r*_cor_=0.76 for Version 3.1 and 0.83 for the updated version) and are attributed to the contamination of the SOHO/SEM signal by flare-related energetic particle fluxes that rise near the end of the 3-h period covered in the correlation. SOHO/SEM is susceptible to signal contamination from protons of energy 10 MeV and greater (Didkovsky *et al.* 2007), yet neither Version 3.1 nor the updated version is corrected for this contamination. Nonetheless, the correlation coefficients for the updated SOHO/SEM are higher than those for Version 3.1 in such cases where both coefficients are abnormally low.

### Comparison of the Daily Average 0.1 – 7 nm and 7 – 50 nm Time Series

The SEM zeroth-order (0.1 – 50 nm) response function remains unchanged from Version 3.1. However, because the algorithm for determining zeroth-order irradiances depends on the first-order measurements [*i.e*. based on the scaling factor *κ* defined in Equation (8)], it is affected by the updates to the first-order response function. Additionally, the weighted mean zeroth-order response function and degradation correction are dependent on the choice of reference spectrum. Thus comparisons between the SOHO/SEM zeroth-order band with SDO/EVE can provide additional validation for these updated data-processing parameters.

**Figure 8:**
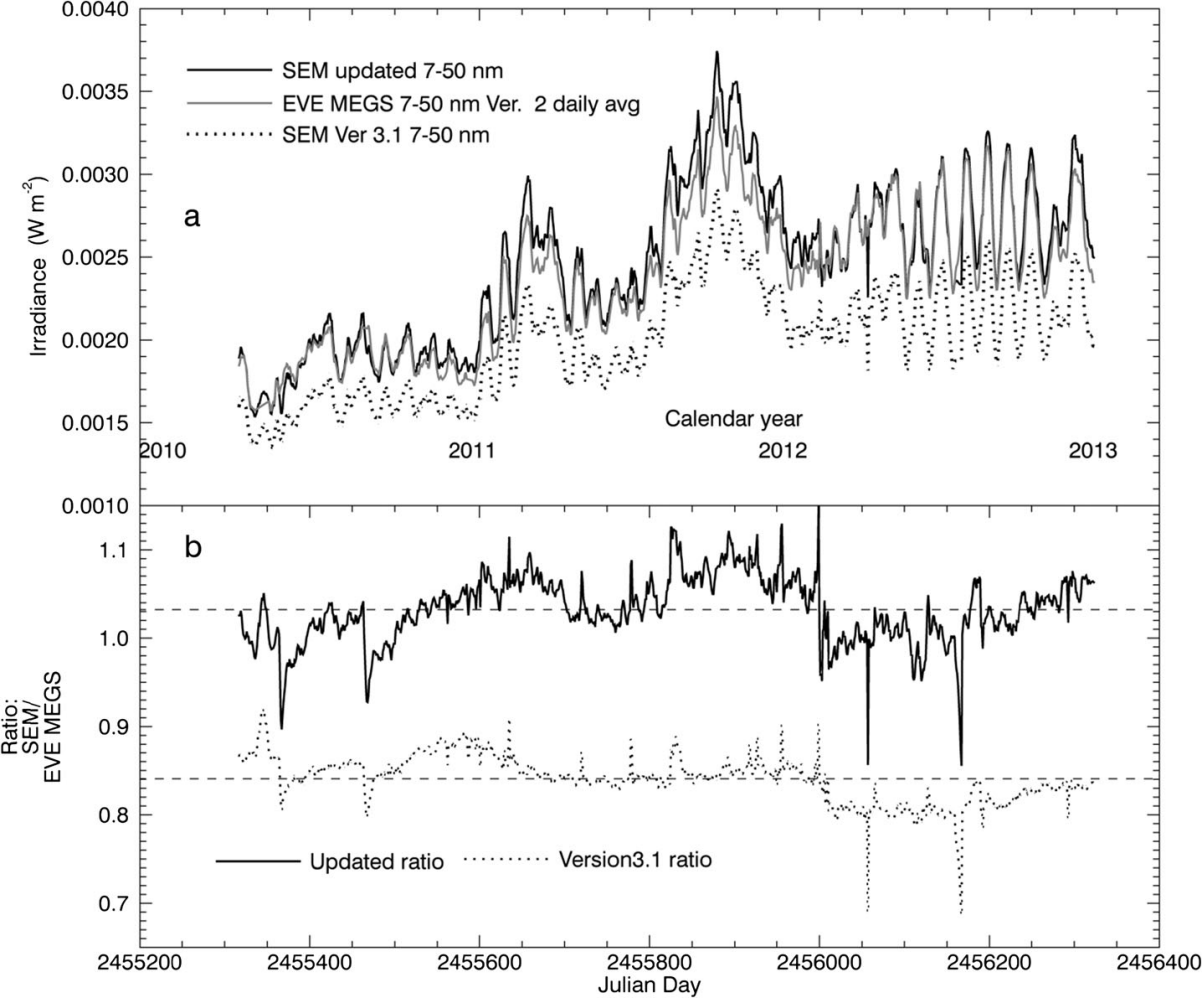
(a) A comparison of the 7 – 50 nm SOHO/SEM irradiance time-series extracted from the zeroth-order measurements for the updated version (black curve) with that for Version 3.1 (dotted curve), and with daily average 7 – 50 nm MEGS integrated spectra. (b) Ratios of the SOHO/SEM Version 3.1 daily average irradiances over the daily average SDO/EVE/MEGS spectra integrated over 7 – 50 nm (dotted line) compared with similar ratios based on the updated SOHO/SEM (solid black line). Mean ratio levels are shown as dashed lines in panel (b).

**Figure 9:**
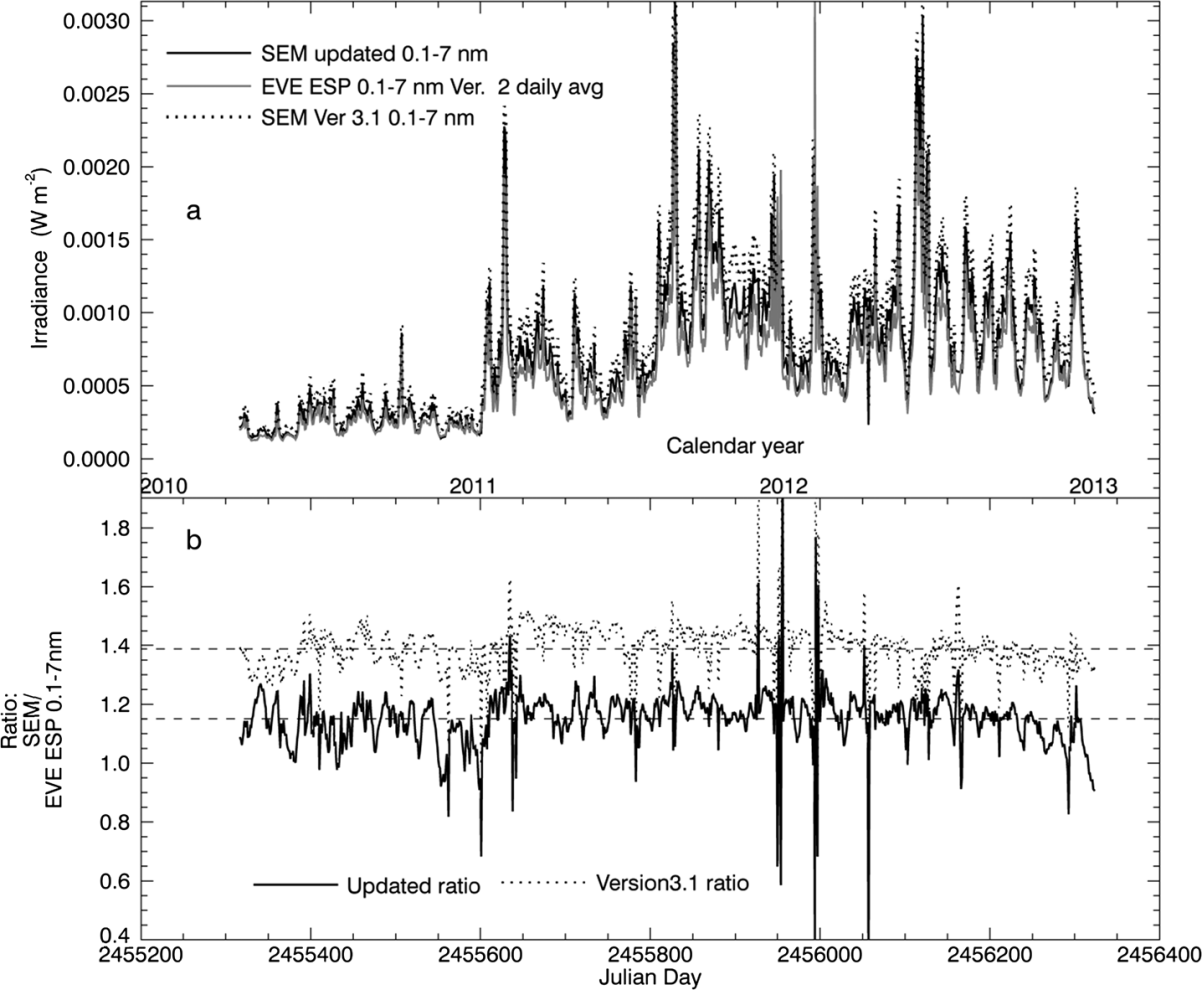
(a) A comparison of the 0.1 – 7 nm SOHO/SEM irradiance time-series extracted from the zeroth-order measurements for the updated version (black curve) with that for Version 3.1 (dotted curve), and with daily average 0.1 – 7 nm ESP measurements. (b) Ratios of the SOHO/SEM Version 3.1 daily average irradiances over the daily average SDO/EVE/ESP 0.1 – 7 nm measurements compared with similar ratios based on the updated SOHO/SEM. Mean ratio levels are shown as dashed lines in panel (b).

None of the individual SDO/EVE channels includes the full 0.1 – 50 nm band measured by the SOHO/SEM zeroth-order channel. However, irradiance within a given portion of the SEM zeroth-order band can be calculated based on the adopted reference spectrum. We calculate irradiance in the 7 – 50 nm band from the SOHO/SEM measurements based on both the updated and Version 3.1 data processing parameters for comparison with integrated MEGS absolute spectra. This comparison is shown in Figure 8. We also compare the 0.1 – 7 nm portion of the SEM zeroth-order band with the EVE/ESP zeroth-order quad diode (QD) channels in Figure 9.

For both of these comparisons SOHO/SEM irradiances agree significantly better with the EVE measurements when calculated using the updated data processing parameters. In the 7 – 50 nm band, the mean ratio for SOHO/SEM over MEGS integrated spectra is about 1.03 for the updated irradiance values compared with about 0.84 for the Version 3.1 values. For the 0.1 – 7 nm comparisons with the ESP QD channels the mean ratio is about 1.15 based on the updated SOHO/SEM irradiances and about 1.4 for Version 3.1. Because the soft X-ray response functions and reference spectra are modeled for both of the SOHO/SEM versions and for the SDO/EVE/ESP QD channel (Didkovsky *et al.* 2012), large differences among data sets are expected in the 0.1 – 7 nm range. For example, Judge *et al.* (2002) demonstrated that the results of derivations of soft X-ray fluxes from SOHO/SEM measurements vary significantly depending on which of two modeled reference spectra is used. This result highlights the need for solar spectral irradiance measurements in the soft X-ray range.

## Conclusions and Application to Future Work

We demonstrate that differences of about 20 % between SOHO/SEM and SDO/EVE measurements of 26 – 34 nm irradiance, which have persisted throughout the SDO mission, can be significantly reduced to about 5 % by applying a revised and more accurate response function, and the now available measured time-dependent reference spectra to the processing of the SEM data. Applying these revised parameters also improves the correlation between high time-cadence SOHO/SEM measurements and simultaneous measurements from SDO/EVE/MEGS during large solar flares. Additionally, comparisons of irradiance in the 0.1 – 7 nm band extracted from SOHO/SEM zeroth-order measurements agree with measurements from SDO/EVE channels within about 15 % on average when the revised parameters are used compared with about 40 % when the Version 3.1 parameters are used.

Recalculation of the entire SOHO/SEM data set, which includes all of solar cycle 23, based on the approach presented here would be of significant value, but would require an alternate time-dependent reference spectrum to use in the absence of SDO/EVE/MEGS measurements. Such a recalculation could, for example, provide verification of, or new information regarding, lower EUV irradiance during the minimum of solar cycles 23/24 compared with that of solar cycles 22/23. Assuming that the solar spectral distribution under solar minimum conditions is consistent from one minimum to the next, it might seem initially that the relative comparison of these two minima should not be affected by whether a time-dependent or fixed reference spectrum is used. However, the same fixed reference spectrum currently used in the SOHO/SEM Version 3.1 data-processing algorithm is also used for processing the sounding-rocket measurements that were performed at different times/activity levels throughout the solar cycle. A change in reference spectrum would affect the 26 – 34 nm irradiance values calculated based on RGIC sounding-rocket measurements differently than those based on SEM clone and SOHO/SEM measurements because the instruments have different response functions. Since the adopted reference spectrum affects the distribution of sounding-rocket measurements, the degradation model based upon them [Equations (1) and (2)] will also be affected.

The alternate reference spectra adopted for recalculating the complete SOHO/SEM data set − whether from an empirical proxy-based model or based on a combination of measurements and physical modeling − should be available for the entire SOHO mission *including the period of overlap with SDO*. Such spectra could then be compared with concurrent MEGS spectra based on the irradiance values obtained when they are used to process the SOHO/SEM data.

## References

[CR1] Acton L.W., Weston D.C., Bruner M.E. (1999). Deriving solar X-ray irradiance from Yohkoh observations. J. Geophys. Res..

[CR2] Bowman B.R., Tobiska W.K., Marcos F., Huang C., Lin C., Burke W. (2008). A new empirical thermospheric density model JB2008 using new solar and geomagnetic indices. AIAA/AAS Astrodynamics Specialist Conference and Exhibit.

[CR3] Carlson R.W., Ogawa H.S., Phillips E., Judge D.L. (1984). Absolute measurement of the extreme UV solar flux. Appl. Opt..

[CR4] Chamberlin P.C., Woods T.N., Eparvier F.G. (2007). Flare irradiance spectral model (FISM): Daily component algorithms and results. Space Weather.

[CR5] Del Zanna G., Bromage B.J.I., Landi E., Landini M. (2001). Solar EUV spectroscopic observations with SOHO/CDS. I. An in-flight calibration study. Astron. Astrophys..

[CR6] Didkovsky L.V., Judge D.L., Jones A.R., Wieman S., Tsurutani B.T., Mcmullin D. (2007). Correction of SOHO CELIAS/SEM EUV measurements saturated by extreme solar flare events. Astron. Nachr..

[CR7] Didkovsky L.V., Judge D.L., Wieman S.R., McMullin D., Cranmer S., Hoeksema T., Kohl J. (2010). Minima of solar cycles 22/23 and 23/24 as seen in SOHO/CELIAS/SEM absolute solar EUV flux. SOHO-23: Understanding a Peculiar Solar Minimum.

[CR8] Didkovsky, L., Judge, D., Wieman, S., Woods, T., Eparvier, F., Jones, A.: Comparison of the first solar EUV irradiance measurements from the SDO/EVE EUV Spectrophotometer (ESP) with the SOHO/CELIAS Solar EUV Monitor (SEM) absolute solar EUV irradiance, 38th COSPAR General Assembly, 2010b E22 – 0016–E22-10.

[CR9] Didkovsky L., Judge D., Wieman S., Woods T., Jones A. (2012). EUV SpectroPhotometer (ESP) in Extreme ultraviolet Variability Experiment (EVE): Algorithms and calibrations. Solar Phys..

[CR10] Dominique M., Hochedez J.-F., Schmutz W., Dammasch I.E., Shapiro A.I., Kretzschmar M., Zhukov A.N., Gillotay D., Stockman Y., BenMoussa A. (2013). The LYRA instrument onboard PROBA2: Description and in-flight performance. Solar Phys..

[CR11] Evans J.S., Strickland D.J., Woo W.K., McMullin D.R., Plunkett S.P., Viereck R.A., Hill S.M., Woods T.N., Eparvier F.G. (2010). Early observations by the GOES-13 Solar Extreme Ultraviolet Sensor (EUVS). Solar Phys..

[CR12] Funsten H.O., Ritzau S.M., Harper R.W., Korde R. (2004). Fundamental limits to detection of low-energy ions using silicon solid-state detectors. Appl. Phys. Lett..

[CR13] Furst M.L., Graves R.M., Madden R.P. (1993). Synchrotron Ultraviolet Radiation Facility (SURF II) radiometric instrumentation calibration facility. Opt. Eng..

[CR14] Haberreiter M., Mandrini C.H., Webb D.F. (2012). Towards the reconstruction of the EUV irradiance for solar cycle 23. Comparative Magnetic Minima: Characterizing Quiet Times in the Sun and Stars.

[CR15] Harrison R.A., Sawyer E.C., Carter M.K., Cruise A.M., Cutler R.M., Fludra A. (1995). The Coronal Diagnostic Spectrometer for the Solar and Heliospheric Observatory. Solar Phys..

[CR16] Henke B.L., Gullikson E.M., Davis J.C. (1993). X-ray interactions: photoabsorption, scattering, transmission, and reflection at *E*=50 – 30 000 eV, *Z*=1 – 92. At. Data Nucl. Data Tables.

[CR17] Hock R.A., Chamberlin P.C., Woods T.N., Crotser D., Eparvier F.G., Woodraska D.L., Woods E.C. (2012). Extreme Ultraviolet Variability Experiment (EVE) Multiple EUV Grating Spectrographs (MEGS): Radiometric calibrations and results. Solar Phys..

[CR18] Hovestadt D., Hilchenbach M., Bürgi A., Klecker B., Laeverenz P., Scholer M. (1995). CELIAS – Charge, Element and Isotope Analysis System for SOHO. Solar Phys..

[CR19] Judge D.L., McMullin D.R., Ogawa H.S., Hovestadt D., Klecker B., Hilchenbach M. (1998). First solar EUV irradiances from SOHO by the CELIAS/SEM. Solar Phys..

[CR20] Judge D., Ogawa H.S., McMullin D.R., Gangopadhyay P., Pap J.M. (2002). The SOHO CELIAS/SEM EUV database from SC23 minimum to the present. Adv. Space Res..

[CR21] Krumrey M., Herrmann C., Müller P., Ulm G. (2000). Synchrotron-radiation-based cryogenic radiometry in the X-ray range. Metrologia.

[CR22] Lean J.L., Emmert J.T., Picone J.M., Meier R.R. (2011). Global and regional trends in ionospheric total electron content. J. Geophys. Res..

[CR23] McMullin D.R., Judge D.L., Hilchenbach M., Ipavich F., Bochsler P., Wurz P., Burgi A., Thompson W.T., Newmark J.S., Pauluhn A., Huber M.C.E., von Steiger R. (2002). In-flight comparisons of solar EUV irradiance measurements provided by the CELIAS/SEM on SOHO. The Radiometric Calibration of SOHO.

[CR24] Nikutowski B., Brunner R., Erhardt Ch., Knecht St., Schmidtke G. (2011). Distinct EUV minimum of the solar irradiance (16 – 40 nm) observed by SolACES spectrometers onboard the International Space Station (ISS) in August/September 2009. Adv. Space Res..

[CR25] Ogawa H.S., Judge D.L. (1986). Absolute solar flux measurement shortward of 575 Å. J. Geophys. Res..

[CR26] Scime E.E., Anderson E.H., McComas D.J., Schattenburg M.L. (1995). Extreme-ultraviolet polarization and filtering with gold transmission gratings. Appl. Opt..

[CR27] Schattenburg M.L., Anderson E.H. (1990). X-ray/VUV transmission gratings for astrophysical and laboratory applications. Phys. Scr..

[CR28] Solomon S.C., Qian L., Didkovsky L.V., Viereck R.A., Woods T.N. (2011). Causes of low thermospheric density during the 2007 – 2009 solar minimum. J. Geophys. Res..

[CR29] Thompson W.T., McMullin D.R., Newmark J.S., Pauluhn A., Huber M.C.E., von Steiger R. (2002). Comparison of CDS irradiance measurements with SEM and EIT. The Radiometric Calibration of SOHO.

[CR30] Tobiska W.K., Bouwer S.D., Bowman B.R. (2006). The development of new solar indices for use in thermospheric density modeling. AIAA/AAS Astrodynamics Specialists Conference and Exhibit.

[CR31] Vest R., Canfield L.R., Furst M.L., Graves R.M., Hamilton A.D., Hughey L.R., Lucatorto T.B., Madden R.P., Carruthers G.R., Dymond K.F. (1999). NIST programs for calibrations in the far ultraviolet spectral region. Ultraviolet Atmospheric and Space Remote Sensing: Methods and Instrumentation II.

[CR32] Wende C.D. (1972). The normalization of solar X-ray data from many experiments. Solar Phys..

[CR33] Wieman S., Judge D.L., Didkovsky L.V., Fineschi S., Fennelly J. (2011). Solar EUV Monitor (SEM) absolute irradiance measurements and how they are affected by choice of reference spectrum. Solar Physics and Space Weather Instrumentation IV.

[CR34] Woods T.N., Rottman G. (2005). The XUV Photometer System (XPS): Solar variations during the SORCE mission. Solar Phys..

[CR35] Woods T.N., Ogawa H., Tobiska K., Farnik F., Pap J.M., Fröhlich C., Ulrich R.K. (1998). SOLERS-22 WG-4 and WG-5 report for the 1996 SOLERS-22 workshop. Solar Electromagnetic Radiation Study for Solar Cycle 22.

[CR36] Woods T.N., Eparvier F.G., Bailey S.M., Chamberlin P.C., Lean J., Rottman G.J., Solomon S.C., Tobiska W.K., Woodraska D.L. (2005). Solar EUV Experiment (SEE): Mission overview and first results. J. Geophys. Res..

[CR37] Woods T.N., Chamberlin P., Eparvier F.G., Hock R., Jones A., Woodraska D. (2012). Extreme ultraviolet Variability Experiment (EVE) on the Solar Dynamics Observatory (SDO): Overview of science objectives, instrument design, data products, and model developments. Solar Phys..

